# Editorial: Age-Based Stereotype Threat Effects on Performance Outcomes

**DOI:** 10.3389/fpsyg.2021.773615

**Published:** 2021-11-02

**Authors:** Hannah J. Swift, Sarah J. Barber, Ruth A. Lamont, David Weiss, Alison L. Chasteen

**Affiliations:** ^1^School of Psychology, University of Kent, Canterbury, United Kingdom; ^2^Department of Psychology, Georgia State University, Atlanta, GA, United States; ^3^College of Medicine and Health, University of Exeter, Exeter, United Kingdom; ^4^Department of Psychology, Leipzig University, Leipzig, Germany; ^5^Department of Psychology, University of Toronto, Toronto, ON, Canada

**Keywords:** age-based stereotype threat, performance, identity, memory, stereotype

## Introduction

The United Nations General Assembly has declared 2021–2030 the decade of healthy aging (World Health Organization, 2021a), but although people are living longer than ever before, ageism and commonly held stereotypes about aging continue to be a risk to healthy aging. Stereotypes about older people can pose a serious challenge for how societies respond to population aging and they can impact adversely on older people's own aging trajectories (Levy, 2009; Swift et al., 2017; World Health Organization, 2021b). Adding to our understanding of the impact of age stereotypes, this special collection of articles focuses on age-based stereotype threat (ABST).

Since its inception in 1995, stereotype threat theory (e.g., Steele and Aronson, 1995) has helped shape the way we understand the impact of negative stereotypes. Stereotype threat is defined as a disruptive concern that arises *in situations* when individuals feel concerned or worried about the possibility of confirming or being judged in light of a negative stereotype. This in turn can result in heightened feelings of anxiety, stress, and worry (Schmader et al., 2008), which can lead to underperformance in task domains associated with the negative stereotype (Steele, 1997), and disengagement and avoidance of these situations or tasks in the future (Woodcock et al., 2012). Hundreds of studies have demonstrated the consequences of stereotype threat, sometimes referred to as *stereotype threat effects* on a range of outcomes and for a variety of negatively stereotyped populations (see Nguyen and Ryan, 2008; Nadler and Clark, 2011; Picho et al., 2013; Appel et al., 2015; Flore and Wicherts, 2015; Gentile et al., 2018 for meta-analytic research on stereotype threat effects).

Early work examining ABST was primarily done within the realm of cognition and focused on how experimental manipulations that highlight (or refute) common assumptions about age-related cognitive decline affect older adults' subsequent cognitive performance (Hess and Hinson, 2006). Subsequent meta-analytic reviews of these ABST studies have revealed reliable decrements for older adults for cognitive (Lamont et al., 2015) and memory performance (Armstrong et al., 2017) when age stereotypes inferring declining cognitive ability are highlighted. However, the magnitude of this effect can be dependent on several moderating factors, including type of experimental manipulation used, chronological age, age-group identification, age-based self-categorization (i.e., seeing the self as “older” rather than “younger”), essentialist beliefs about aging, and regulatory focus, all of which highlight the nuances of stereotype threat effects (Hess and Hinson, 2006; Kang and Chasteen, 2009; Haslam et al., 2012; Barber and Mather, 2013; Lamont et al., 2015; Armstrong et al., 2017; Weiss, 2018).

Beyond cognitive and memory tasks, the second most explored domain for ABST is physical performance, captured via measures of handgrip strength (e.g., Swift et al., 2012), walking (e.g., Chalabaev et al., 2020), and flexibility (Marquet et al., 2018). However, this research has revealed a mixed picture about the impact of age stereotypes on tasks requiring physical or motor action or skill. Some research has revealed significant effects of ABST on physical performance measures (e.g., Swift et al., 2012), while others have found no effect of ABST on physical performance outcomes (e.g., Marquet et al., 2018), or found that there are boundary conditions of ABST on physical tasks, with the effects being dependent on task difficultly and level of self-efficacy/confidence (Barber et al., 2020). These mixed results raise further questions about the nature of ABST, the methods used to capture these effects, and the moderators of the effects, all of which have implications for understanding the application of ABST in everyday contexts where both formal and informal tests of performance emerge (e.g., in employment and healthcare settings; Barber, 2020). This special collection on ABST set out to shed light on some of these issues. We believe the collection of nine articles add to our knowledge in four key areas covering manipulations used to elicit ABST, methodologies used to detect ABST, settings, and people affected by ABST and moderators of ABST.

The present body of work uses a diverse range of methods to explore ABST, including qualitative research (Lamont et al.), quantitative survey research (Gilet et al.; Manzi et al.), and longitudinal survey research (Mariano et al.). The articles within this collection have examined ABST in online (Atkinson et al.), lab-based (Fourquet et al.), and field-based experimental designs (Strickland-Hughes and West). Within the experimental research different types of manipulations are used, including both blatant (Fourquet et al.) and subtle manipulations of ABST (Atkinson et al.) as well as newly defined naturalistic stereotype manipulations explored by Strickland-Hughes and West. For further discussion on experimental manipulations and other methodological insights for ABST effects on memory, the current collection also includes a comprehensive narrative review by Mazerolle et al..

The articles are also diverse in the domains and outcomes they investigate, which range from memory (Mazerolle et al.; Strickland-Hughes and West), meta-memory (Fourquet et al.), emotional recognition (Atkinson et al.), engagement in technology (Mariano et al.), and self-rated performance and organizational relevant variables such as organizational identity and authenticity (Manzi et al.). Manzi et al.'s paper (Manzi et al.) explores the intersection between age and gender stereotype threat on older workers from diverse sample of organizations from Italy, they find that age and gender based stereotypes present a double-threat to women over fifty in the workplace. Lamont et al.'s paper (Lamont et al.) provides further insight into areas of life and domains where younger, middle aged and older adults perceive ABST. Across these articles, moderators are also examined, including educational attainment (Gilet et al.) and participant age (Fourquet et al.; Strickland-Hughes and West), and reviewed (Mazerolle et al.). These studies provide insights into the boundary effects of ABST, which is an issue raised by past research.

Taken together, this special collection both provides further support for the adverse effects of ABST, but also further information on its boundaries and applicability to informal performance contexts. Within these articles there are demonstrations of significant adverse effects of ABST on meta-memory (Fourquet et al.) and increase in false memories for highly educated older adults under ABST (Gilet et al.). Some articles reveal different outcomes of ABST, such as change in organizational identity and self-rated performance (Manzi et al.), and identify new or informal evaluative contexts and age groups that may be subject to ABST and its deleterious effects (Lamont et al.; Mariano et al.). One article also revealed older adult's emotional recognition ability was not impacted by ABST (Atkinson et al.). The majority of the research in the special collection, with the exception of Manzi et al. focuses only on age-identity based stereotype threat, with data collected from predominately White, western contexts (e.g., UK, France, Portugal, Italy, and US).

In the context of global population aging and workforce aging, we believe the research conducted in this special collection of studies make a compelling case that we need to combat negative age stereotypes and change narratives and expectations of aging, as well-inform and educate people so they have the ability to mitigate the influence of age stereotypes. This in turn should reduce vulnerability to negative ABST effects and increase the likelihood of healthy aging. Each article addresses new and important questions regarding the contexts and populations for which ABST effects occur, the strength of ABST effects, and provide a steer for progressing research in this area. We'd like to thank all the authors who contributed to this special collection and we hope you enjoy reading them and learning more about the intricacies of ABST.

## Author Contributions

HS planned, conceptualized, and wrote the first draft of the article. SB, RL, DW, and AC contributed to the main text. All authors contributed to the article and approved the submitted version.

## Conflict of Interest

The authors declare that the research was conducted in the absence of any commercial or financial relationships that could be construed as a potential conflict of interest.

## Publisher's Note

All claims expressed in this article are solely those of the authors and do not necessarily represent those of their affiliated organizations, or those of the publisher, the editors and the reviewers. Any product that may be evaluated in this article, or claim that may be made by its manufacturer, is not guaranteed or endorsed by the publisher.
